# An efficient 3D latent diffusion model for T1-contrast enhanced MRI generation

**DOI:** 10.1088/2057-1976/ae3e96

**Published:** 2026-02-10

**Authors:** Zach Eidex, Mojtaba Safari, Jie Ding, Richard L J Qiu, Justin Roper, David S Yu, Hui-Kuo Shu, Zhen Tian, Hui Mao, Xiaofeng Yang

**Affiliations:** 1 Department of Radiation Oncology, Emory University, Atlanta, GA, United States of America; 2 Winship Cancer Institute, Emory University, Atlanta, GA, United States of America; 3 Department of Radiation & Cellular Oncology, University of Chicago, Chicago, IL, United States of America; 4 Department of Radiology and Imaging Sciences, Emory University, Atlanta, GA, United States of America

**Keywords:** glioma, T1-contrast, rectified flow, intramodal synthesis, deep learning, MRI

## Abstract

Gadolinium-based contrast agents (GBCAs) are commonly employed with T1-weighted (T1w) MRI to enhance lesion visualization but are restricted in patients at risk of nephrogenic systemic fibrosis. In addition, variations in GBCA administration can introduce imaging inconsistencies. This study develops an efficient 3D deep-learning framework to generate T1-contrast enhanced images (T1C) from pre-contrast multiparametric MRI. We propose the 3D latent rectified flow (T1C-RFlow) model for generating high-quality T1C images. First, T1w and T2-FLAIR images are input into a pretrained autoencoder to acquire an efficient latent space representation. A rectified flow diffusion model is then trained in this latent space representation. The T1C-RFlow model was trained on a curated dataset comprised of the Brain Tumor Segmentation (BraTS) 2024 glioma (GLI; 1480 patients), meningioma (MEN; 1141 patients), and metastases (MET; 1475 patients) datasets. Selected patients were split into training (N = 2860), test (N = 614), and validation (N = 612) sets. Model performance was evaluated with the normalized mean squared error (NMSE) and structural similarity index measure (SSIM). Both qualitative and quantitative results demonstrate that the T1C-RFlow model outperforms benchmark 3D models (pix2pix, denoising diffusion probability models (DDPM), Diffusion Transformers (DiT-3D) trained in the same latent space. T1C-RFlow achieved the following metrics - GLI: NMSE 0.044 ± 0.047, SSIM 0.935 ± 0.025; MEN: NMSE 0.046 ± 0.029, SSIM 0.937 ± 0.021; MET: NMSE 0.098 ± 0.088, SSIM 0.905 ± 0.082. In a blinded reader study of 15 patients (5 GLI, 5 MEN, 5 MET), T1C-RFlow received the highest diagnostic-quality scores across all tumor types (3.80 ± 0.45, 3.20 ± 0.45, and 2.60 ± 0.89 on a 5-point Likert scale), significantly outperforming all baseline methods (p < .05). Further studies showed T1C-RFlow to have the best tumor reconstruction performance and significantly faster denoising times (6.9 s volume^−1^, 200 steps) than conventional DDPM models in both latent space (37.7 s, 1000 steps) and patch-based in image space (4.3 h volume^−1^). Our proposed method generates synthetic T1C images that closely resemble radiological features of ground truth T1C in much less time than previous diffusion models. Further development may permit a practical method for contrast-agent-free MRI for brain tumors. Code is made available at https://github.com/zacheidex/An-Efficient-3D-Latent-Diffusion-Model-for-T1-contrast-Enhanced-MRI-Generation.

## 1. Introduction

T1-contrast-enhanced MRI (T1C) is frequently used in oncologic, neural, and vascular imaging workflows where accurate lesion delineation is critical for diagnosis and treatment planning (Ibrahim *et al* 2018, Eidex *et al* 2024a). By injecting a gadolinium-based contrast agent (GBCA), abnormalities such as tumors, inflammation, etc, are significantly enhanced in T1-weighted (T1w) imaging (Roozpeykar *et al* 2022). In oncology, the enhanced tumor is defined as the region with significant contrast uptake on T1C images and is useful in classifying the lesion (Zhou and Lu 2013). However, GBCAs introduce safety concerns (e.g., nephrogenic systemic fibrosis due to gadolinium retention), extend scanning time, and there can be variations in GBCA administrations due to operational complexity and limited availability and affordability in certain resource limiting regions (Iyad *et al* 2023). Therefore, there is a strong clinical interest in developing contrast agent-free alternatives that can capture diagnostic features and information in T1C images.

Although deep-learning methods have made significant progress in generating synthetic T1C (sT1C), key limitations remain. The majority of studies still operate on 2D axial slices which are memory-efficient but cannot provide sufficient through-plane consistency and long-range 3D context (Eidex *et al* 2025a, Li *et al* 2025). Fully 3D networks improve anatomical coherence but rely on patch-based training and sliding-window inference to fit GPU memory, so long-range relationships are not well captured (Pan *et al* 2024, Shaoyan *et al* 2025). Furthermore, constructing the sT1C volume from patches not only leads to long prediction times but also can introduce overlap-stitching and boundary artifacts. (Bieder *et al*^2024^).

Denoising diffusion probability models (DDPMs) (Ho *et al* 2020, Chang *et al* 2024) are the current state-of-the-art method over generational adversarial networks (GANs) like pix2pix (Isola *et al*^2017^) since they deliver strong fidelity without the training stability challenges of GANs. (Becker *et al* 2022) However, DDPMs require one thousand steps to reconstruct the image from noise and input priors (T1w and T2-FLAIR MRI), so high-resolution inference times can take several hours, limiting applicability to clinical practice. To overcome this issue, latent diffusion models (LDMs) (Rombach *et al* 2022) first compress the input volumes into a compressed latent space representation, so the computational requirements and inference times are significantly lessened. Furthermore, advanced noise schedulers like the denoising diffusion implicit model (DDIM) (Song *et al* 2021), pseudo numerical methods for diffusion model (PNDM) (Liu *et al* 2022a), and rectified flow (RFlow) (Esser *et al* 2024) schedulers reduce the inference timesteps from 1000 to 300 or fewer timesteps. These advances enable diffusion models to be efficiently used in clinical practice and allow for more advanced architectures such as Diffusion Transformers (DiT-3D) (Mo *et al* 2023, Peebles and Xie 2023) by reducing computational requirements. In this paper, we propose the 3D latent rectified flow (T1C-RFlow) model for the prediction of tumor-specific T1-constrast enhancement from multiparametric (T1w and T2-FLAIR) volumes. We perform the training in a compressed latent space representation and implement the rectified flow noise scheduler, improving performance while reducing the computation requirements for training and inference.

The impact and contributions of this paper can be summarized as follows:

• Model training and inference are performed in the latent space of Monai Medical AI for Synthetic Imaging’s (MAISI’s) pretrained 3D variational autoencoder (VAE) (Guo *et al* 2025), reducing the spatial dimensions from 1 × 256 × 256 × 192 to 4 × 64 × 64 × 48 and allowing the entire volume to be captured by the network.
• An RFlow noise scheduler is used which outperforms DDPM in fewer timesteps (200 versus 1000 timesteps). Our model’s denoising time (6.9 s volume^−1^, 200 steps) is significantly reduced compared to conventional 3D DDPM models in both latent space (37.7s, 1000 steps) and patch-based in image space (4.3 h volume^−1^).
• We train our model on T1w and T2-FLAIR images from 3 Brain Tumor Segmentation (BraTS) 2024 challenge datasets (de Verdier *et al* 2024) - glioma (GLI; 1480 patients), meningioma (MEN; 1141 patients), and metastases (MET; 1475 patients), so that our model is generalizable to several disease types.

## 2. Methods

### 2.1. Data acquisition and preprocessing

A multi-institutional cohort was assembled from the BraTS 2024 challenge (de Verdier *et al* 2024) comprised of glioma (GLI; 1,480), meningioma (MEN; 1,141), and metastasis (MET; 1,475) cases with co-registered T1w, T2-FLAIR, and T1C volumes. The provided MRI volumes were converted from DICOM to NIfTI format and skull stripped using HD-BET (Isensee *et al* 2019). The brain-extracted T1, T1C, T2, and T2-FLAIR sequences were coregistered to the Linear Symmetrical MNI Atlas with affine registration. Additionally, we resampled all scans to 1 mm isotropic resolution (256 × 256 × 192 voxels). Intensities were normalized to [−1,1] across the input volume. We randomly split subjects into train, test, and validation splits of 2860, 614, and 612 patients respectively while maintaining the relative proportions of GLI, MEN, and MET cases in each split.

### 2.2. VAE10.48550/arXiv.2502.14064

A VAE learns an encoder q_*φ*_(z|x) and decoder p_*θ*_($\hat{x}$|z) over a latent variable, z, with a simple prior p(z) = ${\mathscr{N}}$(**0**, **I**) (Kingma and Welling 2022). The encoder maps an input x to the parameters of a diagonal-Gaussian posterior, *μ*(x) and *σ*(x). A latent sample is then drawn via the reparameterization trick

\begin{eqnarray*}\begin{array}{c}z=\mu \left({\boldsymbol{x}}\right)+\sigma \left({\boldsymbol{x}}\right)\varepsilon ,\,\varepsilon \unicode{x0007E}N\left(0,{\boldsymbol{I}}\right)\end{array}\end{eqnarray*}

which enables low-variance gradient estimates. The decoder reconstructs $\hat{x}$ from z. VAEs are trained by maximizing the evidence lower bound (ELBO), combining a reconstruction term with a Kullback-Leibler (KL) divergence that regularizes q_*φ*_(z|x) toward the Gaussian prior, improving stability for latent-space transport methods (Asperti and Trentin 2020).

We employ MAISI’s pretrained 3D VAE to obtain compact volumetric latents for each sequence (T1w, T2-FLAIR, T1C). (Guo *et al*) Inputs are passed through a UNet-style encoder that yields two 4-channel tensors ***μ*****(x)** and ***σ*****(x)** $\in $ ℝ^4 × 64 × 64 × 48^ from a 256 × 256 × 192 volume (i.e., 1/4 spatial resolution per axis; 64 ×  fewer voxels). Stochastic latents are formed via (1), and we precompute ***μ*** and ***σ*** before training. During training, the T1w and T2-FLAIR conditioning latents are concatenated channelwise, while the T1C target latent serves as the ground truth. During inference, the sT1C latent is decoded by the fixed decoder to reconstruct a full-resolution 3D T1C volume. Operating in this low-dimensional latent space yields substantial time savings and allows the entire 3D volume to be processed at once during inference, in contrast to 3D patch-based image-space approaches, while still preserving anatomical details of the tumor region.

To assess whether a task-specific autoencoder would further improve performance, we also attempted to fine-tune MAISI’s VAE on our BraTS cohort. However, we found that fine-tuning consistently degraded the visual fidelity of the reconstructions and slightly worsened quantitative reconstruction metrics compared to the original pretrained weights. We therefore used the publicly released MAISI VAE without additional task-specific adaptation in all experiments.

### 2.3. Rectified flow diffusion U-net

RFlow casts generation as learning a time-conditioned velocity field ${v}_{\theta }\left(z,t,c\right)$ that transports a simple noise distribution to the data distribution along a deterministic ordinary differential equation (Liu *et al* 2022b) Unlike score-based DDPMs, which require stochastic reverse-time integration with many denoising steps, RFlow learns the drift directly, enabling deterministic sampling with substantially fewer steps (Lee *et al* 2024). Let ${z}_{0}$ denote a clean latent (T1C target) and ${z}_{1}\sim {\mathscr{N}}\left(0,{\boldsymbol{I}}\right)$ denote a noise latent. With a monotone schedule $\alpha \left(t\right)$ such that $\alpha \left(0\right)=0$ and $\alpha \left(1\right)=1$, we define the linear path (2) and the corresponding ground-truth velocity (3):

\begin{eqnarray*}{z}_{t}=\left(1-\alpha \left(t\right)\right)\,{z}_{0}+\alpha \left(t\right)\,{z}_{1}\end{eqnarray*}

\begin{eqnarray*}{v}^{* }\left({z}_{t},t\right)=\displaystyle \frac{d}{dt}{z}_{t}=\alpha ^{\prime} \left(t\right)\,\left({z}_{1}-{z}_{0}\right)\end{eqnarray*}

**Figure 1.**
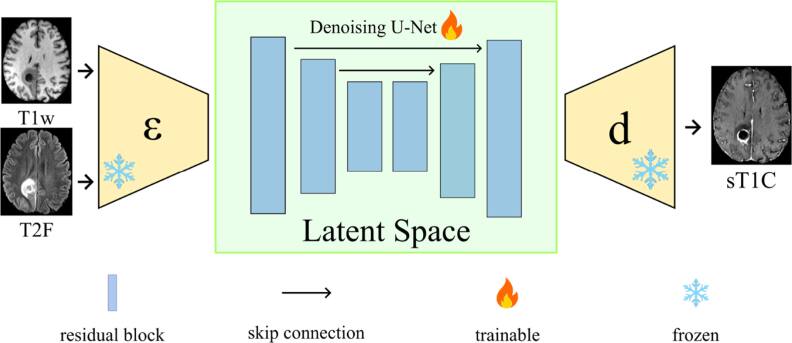
Schematic flow diagram of T1C-RFlow. MAISI’s pretrained autoencoder compresses and decompresses the input T1w and T2-FLAIR images latent space representations. These latents together with a timestep embedding and noised vector are input into a rectified flow denoising U-Net which is trained to output the corresponding T1C latent.

We train a 3D diffusion UNet with a symmetrical encoder and decoder containing [128, 128, 256] channels at each layer and 2 residual blocks per layer to approximate ${v}_{\theta }\left({z}_{t},t,c\right)$ with an ${{\mathrm{l}}}_{1}$ transport loss against the analytical target above, using timesteps drawn from the RFlow scheduler (1000 training timesteps) and conditioning vector, $c$, formed by channel-wise concatenation of the T1w and T2-FLAIR latents (figure 1). The network input is the concatenation, $\left[\,{z}_{t}\left|\left|{z}_{{\mathrm{T1W}}}\right|\right|{z}_{{\mathrm{FLAIR}}}\,\right]$ (12 × 64 × 64 × 48) where $| | $ denotes the channel-wise concatenation operator. Sampling integrates the learned ODE deterministically from t = 1 to t = 0 using a fixed-step solver with K steps (default K = 200):

\begin{eqnarray*}{ {\mathcal L} }_{RFLOW}\left(\theta \right)={E}_{{z}_{0},{z}_{1},t}\left[| | {v}_{\theta }\left({z}_{t},t,c\right)-{v}^{* }\left({z}_{t},t\right)| {| }_{1}\right]\end{eqnarray*}

\begin{eqnarray*}\displaystyle \frac{dz}{dt}={v}_{\theta }\left(z,t,c\right),\,z\left(1\right)\sim {\mathscr{N}}\left(0,{\boldsymbol{I}}\right),\,t:1\to 0\end{eqnarray*}

\begin{eqnarray*}\begin{array}{c}{z}^{k-1}={z}^{k}-{\mathrm{\Delta }}t\,{v}_{\theta }\left({z}^{k},{t}_{k},c\right),\\ \,k=K,\ldots ,\,1,\,{\mathrm{\Delta }}t=\displaystyle \frac{1}{k}\end{array}\end{eqnarray*}

### 2.4. Implementation details

The T1C-RFlow model and competing methods were trained with the AdamW optimizer (Loshchilov and Hutter 2019) (learning rate 1 × 10^–5^, *β*_1_ = 0.9, *β*_2_ = 0.999, weight-decay 1 × 10^–4^) for 100 epochs with a batch size of 4 on a single NVIDIA A6000 ADA (48 GB). The rectified flow scheduler implemented 1000 training timesteps and a logit-normal sampling distribution (Esser *et al*) based on the latent size (64 × 64 × 48) with 3 spatial dimensions. Competing methods (DDPM and Pix2pix) were trained according to the hyperparameters and learning schedule as proposed in the original works (Ho *et al* 2020, Isola *et al*^2017^). The DiT-3D model was modified to work on medical images as opposed to point clouds in the original implementation (Mo *et al* 2023) and to use a rectified flow noise scheduler. During inference, T1C-RFlow and DiT-3D both used 200 timesteps while DDPM used 1000 timesteps. The generated latents from all methods were then translated to image space with the VAE’s decoder for evaluation. The full implementation can be found at https://github.com/zacheidex/An-Efficient-3D-Latent-Diffusion-Model-for-T1-contrast-Enhanced-MRI-Generation

### 2.5. Validation and evaluation

We evaluated the final performance on the test set (N = 614) of the holdout test. All methods were evaluated on the full 3D decoded image-space volumes normalized from [−1,1]. For each patient, quantitative accuracy was computed over the whole MRI volume and summarized as mean ± std across the test cohort and further stratified by disease type (GLI; N = 239, MEN; N = 162, MET; N = 213). We report normalized mean-squared error (NMSE) (Safari *et al* 2024), peak signal-to-noise ratio (PSNR) (Safari *et al* 2025), normalized cross-correlation (NCC) (Qiu *et al* 2021), and structural similarity (SSIM) (Eidex *et al* 2024b). The SSIM was formulated with the stabilizing constants, C_1_ and C_2_, set to C_1_ = (0.01$L$)^2^ and $C$_2_ = (0.03$L$)^2^ where L is the dynamic range (L = 2). In addition to calculating metrics for the whole brain volume, we also calculated metrics for the tumor region. We defined the tumor region by creating a bounding box around the provided segmentation maps to define a bounding box with a padding of 5 voxels and calculated the metrics within this bounding box. Since some segmentation maps were not provided, a subset of 545 patients with segmentation maps was used to evaluate the tumor region. Since NMSE quantifies the normalized residual energy and increases monotonically with error (lower is better); PSNR restates the mean-squared error on a logarithmic decibel scale relative to the dynamic range and tends to align with perceived fidelity (higher is better). NCC measures linear agreement in structure while being insensitive to global intensity shifts and global scaling (higher is better); and SSIM evaluates luminance, contrast, and structural similarity to better align with human perception (higher is better).

For statistical analysis, per-patient metric values were compared using a two-sided Welch’s t-test (unequal variances) within each cohort (overall and across each dataset) (Delacre *et al* 2017). A significance level of *α* = .05 and p < .05 was considered statistically significant.

To assess visual fidelity, we performed a blinded reader study on 15 patients from the test set (5 GLI, 5 MEN, and 5 MET patients). Two board certified clinicians with experience in radiation oncology independently rated the diagnostic quality of each synthetic T1C image on a five-point Likert scale (table 1) compared to the ground truth. For each test case and method, the scores from the two readers were averaged to obtain a per-case mean score. Statistical comparisons between T1C-RFlow and each baseline method were performed using a two-sided paired t-test applied to these per-case mean scores within each disease cohort.

**Table 1.** Blinded clinical scoring grading criteria.

Score	Description
1	Non-acceptable: strong artifacts, image not usable for clinical decision making.
2	Poor but diagnostic: substantial artifacts; interpretation possible but limited.
3	Acceptable: some kind of artifacts only, but adequate for routine diagnosis.
4	Very good: minor artifacts only; adequate for routine diagnosis.
5	Excellent: no or negligible artifacts; fully diagnostic quality.

## 3. Results

The proposed T1C-RFlow model demonstrated superior performance in synthesizing T1C MRI compared to existing state-of-the-art methods. The T1C-RFlow model achieved the highest fidelity metrics for whole brain volumes on the test set, with noticeably improved mean NMSE, PSNR, and NCC (table 2). In particular, the proposed method improved average NMSE by 25% compared to the next best method (DDPM). All improvements were statistically significant except for SSIM when compared to pix2pix. These results remained consistent when stratified by dataset (GLI, MEN, and MET) although all methods produced quantitatively worse sT1C for metastases compared to glioma and meningioma. This may be due to lower quality T1C images in this dataset. For the tumor region, T1C-RFlow again had the highest performance on the evaluation metrics with most differences being statistically significant (p < .05). The GLI dataset contained the most heterogeneous, complex tumors, which may account for why all methods had the lowest tumor-region SSIM and NCC values in this cohort (table 3). In addition to quantitative metrics, the blinded clinical scoring study corroborated these findings (table 4). Across GLI, MEN, and MET cohorts, T1C-RFlow achieved the highest diagnostic-quality ratings, with mean scores of 3.80 ± 0.45, 3.20 ± 0.45, and 2.60 ± 0.89, respectively, corresponding to acceptable image quality. All differences between T1C-RFlow and baseline methods were statistically significant within each disease group (p < .05), while the baseline models were frequently rated as poor or non-acceptable quality. These reader scores indicate that the quantitative gains of T1C-RFlow translate into improved perceived diagnostic utility from a clinical perspective.

**Table 2.** Quantitative performance for the whole brain volume of the proposed method compared to state-of-the-art methods for the GLI, MEN, and MET datasets as well as averaged together. All methods are 3D and trained in the same latent space as the proposed method. P-value metrics are compared against T1C-RFlow’s performance.

		NMSE[↓]		PSNR (dB) [↑]		NCC[↑]		SSIM[↑]	
		Mean ± Std.	p-value	Mean ± Std.	p-value	Mean ± Std.	p-value	Mean ± Std.	p-value
**All**	Proposed (T1C-RFlow)	**0.063** ± **0.067**	—	**29.7** ± **3.0**	—	**0.957** ± **0.018**	—	**0.925** ± **0.054**	—
	DDPM	0.084 ± 0.078	<.001	28.3 ± 3.0	<.001	0.951 ± 0.021	<.001	0.903 ± 0.059	<.001
	Pix2pix	0.091 ± 0.250	0.007	28.4 ± 2.9	<.001	0.933 ± 0.030	<.001	**0.923** ± **0.051**	0.546
	DiT-3D	0.118 ± 0.074	<.001	26.5 ± 3.0	<.001	0.922 ± 0.030	<.001	0.874 ± 0.077	<.001

GLI	Proposed (T1C-RFlow)	**0.044** ± **0.047**	—	**29.9** ± **2.9**	—	**0.960** ± **0.015**	x	**0.935** ± **0.025**	—
	DDPM	0.064 ± 0.045	<.001	28.2 ± 2.9	<.001	0.957 ± 0.016	0.036	0.910 ± 0.041	<.001
	Pix2pix	0.057 ± 0.041	0.002	28.7 ± 2.7	<.001	0.942 ± 0.029	<.001	0.931 ± 0.027	0.109
	DiT-3D	0.096 ± 0.055	<.001	26.4 ± 2.9	<.001	0.928 ± 0.028	<.001	0.885 ± 0.047	<.001

MEN	Proposed (T1C-RFlow)	**0.046** ± **0.029**	—	**29.9** ± **2.7**	—	**0.961** ± **0.014**	—	**0.937** ± **0.021**	**—**
	DDPM	0.064 ± 0.037	<.001	28.3 ± 2.7	<.001	0.956 ± 0.018	0.005	0.913 ± 0.035	<.001
	Pix2pix	0.058 ± 0.034	<.001	28.7 ± 2.5	<.001	0.942 ± 0.018	<.001	0.936 ± 0.022	0.45
	DiT-3D	0.104 ± 0.062	<.001	26.2 ± 2.8	<.001	0.930 ± 0.023	<.001	0.881 ± 0.053	<.001

MET	Proposed (T1C-RFlow)	**0.098** ± **0.088**	—	**29.4** ± **3.3**	—	**0.949** ± **0.020**	—	0.905 ± 0.082	—
	DDPM	0.122 ± 0.111	0.013	28.3 ± 3.2	<.001	0.941 ± 0.024	<.001	0.886 ± 0.084	0.02
	Pix2pix	0.155 ± 0.412	0.05	27.9 ± 3.4	<.001	0.918 ± 0.033	<.001	**0.905** ± **0.075**	0.94
	DiT-3D	0.154 ± 0.085	<.001	26.8 ± 3.3	<.001	0.911 ± 0.032	<.001	0.855 ± 0.110	<.001

**Table 3.** Quantitative performance for the tumor region of the proposed method compared to state-of-the-art methods for the GLI, MEN, and MET datasets as well as averaged together.

		NMSE[↓]		PSNR (dB) [↑]		NCC[↑]		SSIM[↑]	
		Mean ± Std.	—	Mean ± Std.	p-value	Mean ± Std.	p-value	Mean ± Std.	p-value
All	Proposed (T1C-RFlow)	**0.074** ± **0.054**	—	**21.7** ± **5.8**	—	**0.486** ± **0.290**	—	**0.570** ± **0.170**	—
	DDPM	0.126 ± 0.566	0.035	20.4 ± 5.0	<.001	0.435 ± 0.302	0.004	0.504 ± 0.180	<.001
	Pix2pix	0.135 ± 0.610	0.021	20.0 ± 5.5	<.001	0.403 ± 0.310	<.001	0.549 ± 0.171	0.039
	DiT-3D	0.151 ± 0.169	<.001	18.5 ± 4.8	<.001	0.309 ± 0.301	<.001	0.438 ± 0.166	<.001

GLI	Proposed (T1C-RFlow)	**0.090** ± **0.047**	—	**19.5** ± **2.9**	—	**0.282** ± **0.196**	—	**0.462** ± **0.127**	—
	DDPM	0.126 ± 0.116	<.001	18.6 ± 2.9	0.001	0.219 ± 0.210	0.001	0.407 ± 0.124	<.001
	Pix2pix	0.113 ± 0.070	<.001	18.7 ± 2.9	0.007	0.194 ± 0.232	<.001	0.476 ± 0.137	0.274
	DiT-3D	0.162 ± 0.092	<.001	17.1 ± 3.4	<.001	0.122 ± 0.185	<.001	0.389 ± 0.116	<.001

MEN	Proposed (T1C-RFlow)	**0.061** ± **0.058**	—	**24.8** ± **8.7**	—	**0.658** ± **0.277**	—	**0.699** ± **0.182**	—
	DDPM	0.083 ± 0.067	0.004	23.0 ± 7.2	0.057	0.604 ± 0.291	0.106	0.641 ± 0.202	0.011
	Pix2pix	0.091 ± 0.121	0.007	23.2 ± 8.2	0.115	0.623 ± 0.271	0.271	0.672 ± 0.191	0.223
	DiT-3D	0.130 ± 0.100	<.001	20.7 ± 6.7	<.001	0.491 ± 0.307	<.001	0.564 ± 0.208	<.001

MET	Proposed (T1C-RFlow)	**0.067** ± **0.054**	—	**21.8** ± **4.1**	—	**0.589** ± **0.248**	—	**0.596** ± **0.119**	—
	DDPM	0.159 ± 0.957	0.193	20.5 ± 3.6	<.001	0.554 ± 0.247	0.172	0.509 ± 0.142	<.001
	Pix2pix	0.195 ± 1.032	0.093	18.9 ± 4.1	<.001	0.474 ± 0.263	<.001	0.538 ± 0.132	<.001
	DiT-3D	0.154 ± 0.257	<.001	18.4 ± 3.7	<.001	0.383 ± 0.285	<.001	0.398 ± 0.126	<.001

**Table 4.** Blinded clinical scoring assessing T1C-RFlow’s diagnostic quality compared to state-of-the-art methods for the GLI, MEN, and MET datasets five-point scale where 1 is non-acceptable quality and 5 is excellent, fully diagnostic quality. Values are reported as mean ± standard deviation.

	GLI		MEN		MET	
	Mean ± Std.	p-value	Mean ± Std.	p-value	Mean ± Std.	p-value
Proposed (T1C-RFlow)	**3.80** ± **0.45**	—	**3.20** ± **0.45**	—	**2.60** ± **0.89**	—
DDPM	2.40 ± 0.55	0.005	1.20 ± 0.45	0.003	1.00 ± 0.00	0.016
Pix2pix	2.20 ± 0.84	0.003	1.80 ± 0.45	0.005	1.40 ± 0.55	0.033
DiT-3D	1.00 ± 0.00	<.001	1.00 ± 0.00	<.001	1.00 ± 0.00	0.016

**Figure 2.**
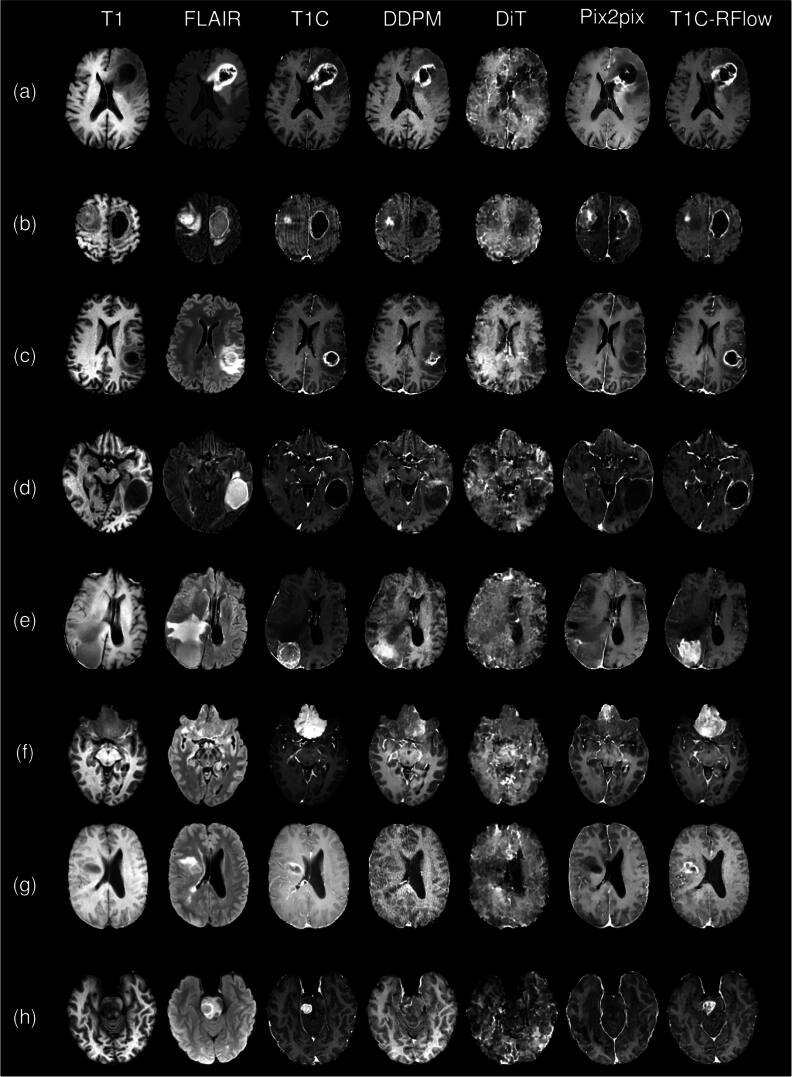
Example sT1C compared to ground truth slices generated from DDPM, DiT, Pix2pix, and T1C-RFlow together with the input T1w and T2-FLAIR MRIs. Rows (a)–(d) are glioma (BraTS-GLI); rows (e) and (f) are meningioma (BraTS-MEN); rows (g) and (h) are metastases (BraTS-MET).

We also compared model inference time and GPU usage on an A6000 ADA GPU (table 5) and found the total time of the T1C-RFlow model to be a reasonable 9.7 s (1.2 s encode + 6.9 s denoising + 1.6 s decoding) especially compared to conventional 3D patch-based DDPM methods (4.3 h). Pix2pix had the fastest inference time since the latent prediction happens in a single step compared to 200 steps for T1C-RFlow and 1000 steps for the tested DDPM model. However, sT1C generated by pix2pix was suboptimal in terms of the image similarity/quality metrics. Compressing the images with a VAE resulted in minimal visual differences and was verified quantitively with the T1w, T2-FLAIR, and T1-contrast volumes having PSNR values near 35 dB (table 6). Finally, we found that a multimodal approach (T1w + T2-FLAIR) was significantly better (p < .01) than T1w or T2-FLAIR alone by running model inference with and without zeroing the input tensors from each sequence (table 7). Visually, the sT1C images generated by T1C-RFlow are closest to the real contrast-enhanced scans, although regions with contrast show slight visual differences and the overall brightness of T1C and sT1C of the proposed method can be very different especially with low quality ground truth T1C scans (figure 2; rows (e)–(g)). T1C-RFlow outperforms competing methods for glioma (figure 2; Rows (a)–(d)) meningioma (figure 2; rows (e) and (f)) and metastases (figure 2; rows (g) and (h)).

**Table 5.** Inference times and maximum GPU usage on an A6000 ADA GPU. The proposed method takes less than 10 seconds per volume compared to over 4 h using a conventional patch based DDPM diffusion model (patch size 64 × 64 × 48). Max GPU usage is measured at inference.

	Timesteps	Inference Time	Max GPU Usage (GB)
Autoencoder (Encode)	—	1.2 s	7.6
Autoencoder (Decode)	—	1.6 s	12.5
Pix2pix	—	74.0 ms	12.5
DDPM (Latent)	1000	37.7 s	12.5
DDPM (Patches)	1000	4.3 h	1.3
DiT-3D	200	11.9 s	13.1
Proposed (T1C-RFlow)	200	6.9 s	15.2

**Table 6.** Quantitative performance of MAISI’s VAE ability to accurately encode and decode T1w, T2-FLAIR, and T1C sequences compared to the original input image.

Modality	NMSE(×10^–2^) [↓]	PSNR (dB) [↑]	SSIM[↑]
T1w	0.63 ± 0.25	35.3 ± 2.0	0.979 ± 0.006
T2-FLAIR	1.11 ± 0.58	34.8 ± 1.9	0.972 ± 0.008
T1C	1.63 ± 1.11	35.2 ± 1.5	0.973 ± 0.008

**Table 7.** Ablation studies of the proposed method with T1w, T2-FLAIR, and both T1w and T2-FLAIR MRI. Our model achieved the best performance using information from both sequences together.

	NMSE[↓]		PSNR (dB) [↑]		NCC[↑]		SSIM[↑]	
	Mean ± Std.	p-value	Mean ± Std.	p-value	Mean ± Std.	p-value	Mean ± Std.	p-value
T1w + T2-FLAIR	**0.063** ± **0.067**	—	**29.7** ± **3.0**	—	**0.957** ± **0.017**	—	**0.925** ± **0.054**	—
T1w only	0.074 ± 0.071	0.008	29.0 ± 3.0	<.001	0.946 ± 0.024	<.001	0.916 ± 0.054	0.004
T2-FLAIR only	0.117 ± 0.079	<.001	26.4 ± 2.5	<.001	0.892 ± 0.034	<.001	0.881 ± 0.060	<.001

## 4. Discussion

In this study, we demonstrate the T1C-RFlow model quantitatively outperforms the state-of-the art DDPM, the GAN-based Pix2pix, and diffusion-transformer-based DiT-3D models while generating sT1C volumes in less than 10 seconds. By training our model using T1w and T2-FLAIR images from 3 Brain Tumor Segmentation (BraTS) 2024 challenge datasets (de Verdier *et al* 2024) (GLI, MEN, and MET) we make our model generalizable to several disease types. Further development of our approach may permit a practical method for capturing the entire 3D image context for contrast-agent-free MRI for brain tumors, eliminating GBCA-associated risk and simplifying clinical protocols. We use a 3D LDM for synthesizing T1C from multiparametric MRI. Conventional 2D slice-by-slice models struggle to capture volumetric context leading to inconsistent enhancement across slices. Patch-based 3D models must similarly limit their context to a portion of the MRI volume and incorporate computationally expensive overlapping tile strategies to avoid visual artifacts due to discrepancies between tiles. Our 3D latent diffusion framework (T1C-RFlow) addresses these issues by operating in a compressed latent space, which drastically reduces memory requirements and captures the entire 3D volume at once.BIEDER

Our design builds upon insights from recent works. Eidex *et al* improved synthetic T1C quality using a vision Transformer (ViT) conditioned on tumor segmentation maps (Eidex *et al* 2025a). Ma *et al* leveraged a 2D latent diffusion model with ControlNet guidance to synthesize T1C from T2-FLAIR, achieving superior image quality and aiding tumor delineation (Ma *et al* 2025). In addition, Li *et al* showed the advantages of a multiparametric transformer-based approach and integrating context from the tumor region (Li *et al* 2025). However, all studies were designed for 2D axial slices and may struggle with long-range 3D context. In addition, these studies did not investigate generalizability to different disease types.

Qualitatively, our method captures fine anatomical details and the contrast-enhancing tumor region much more faithfully than baseline methods (table 3) and may even surpass or restore detail to low-quality ground truth T1C volumes (e.g. patient motion, artifacts, variability in contrast agent administration) (figure 2). The blinded reader study further supports these qualitative observations, with T1C-RFlow rated as having acceptable diagnostic quality for all three tumor types, while baseline methods were more frequently scored as poor or non-acceptable (table 4). We identify several factors that may have affected this result. Compared to GAN-based models like pix2pix, diffusion models have demonstrated improved training stability and image quality at the cost of increased inference time. In addition, compared to the original DDPM formulation, rectified flow methods often converge more quickly and produce higher quality results than DDPM in fewer timesteps by traversing a more direct path from noised to target latent space. Although powerful, 3D ViT-based methods must compress the 4D source latent into a 1D tensor of image patches before performing the self-attention operation (Dosovitskiy *et al* 2021, Eidex *et al* 2025b). The spatial relationships are retained through learnable position embeddings, but this task can be challenging and lead to unstable training.

Despite these advantages, synthesizing contrast uptake in pathological regions remains intrinsically challenging often resulting in low agreement with ground truth T1C volumes. Tumor regions occupy a small fraction of the brain volume, so voxel-wise losses and global metrics are dominated by normal tissue and can favor conservative, slightly over-smoothed predictions in the lesion. In addition, gliomas in the BraTS-GLI cohort are the most heterogeneous and infiltrative tumors, exhibiting necrotic cores, irregular ring enhancement, and ill-defined margins that make inference uniquely difficult to infer from pre-contrast T1w and T2-FLAIR inputs (table 3). Small spatial misalignments or local intensity differences at the enhancing margin can further disproportionately reduce SSIM and NCC even when the overall image remains clinically acceptable. Potential solutions to further improve tumor-region fidelity include incorporating explicit tumor information (e.g., segmentation maps or predicted lesion probability maps) as additional conditioning inputs, designing tumor-aware loss functions that up-weight errors within the enhancing lesion (Wu *et al* 2023) and adding lightweight post-processing or hybrid modules that refine contrast and edges in the tumor region while preserving the globally consistent sT1C volume produced by T1C-RFlow. However, outside of post-processing, the pretrained VAE was not trained on segmentation maps, so finetuning the VAE on this information is the first step in implementing these approaches.

We note several limitations of this study. First, our study was limited to brain tumor cohorts from the BraTS 2024 challenge, so performance on other neurological diseases or imaging protocols remains to be validated. Second, while our model implicitly captured tumor enhancement patterns, it did not use explicit tumor masks or structural priors during training. While technically possible, this was avoided since MAISI’s pretrained VAE was not trained directly on segmentation maps so would require extensive finetuning to accurately reconstruct the segmentation maps. This may help in difficult cases with large, heterogeneous tumor volumes. In addition, our convolution-based architecture did not allow for a straightforward analysis or visualization of feature interpretability. Scaling T1C-RFlow toward a foundation model trained on larger, multi-institutional datasets could further improve robustness and generalizability (Wang *et al* 2025a, 2025b).

Future work will investigate developing and externally validating a foundation model that jointly generates multiple MR sequences (rather than only T1-contrast) on a larger, multi-institutional multimodal dataset, with the goal of improving robustness and facilitating domain adaptation across scanners, institutions, and acquisition protocols. We are also interested in achieving higher performance with ViT, Swin transformer, (Liu *et al* 2021, Pan *et al* 2024) and highly optimized convolution based models which can situationally outperform ViT models (Liu *et al* 2022). These ViT based models additionally offer improved interpretability and feature visualization through their use of attention maps. Finally, we are confident that integrating segmentation map information, either directly or by prediction, into the sT1C generation pipeline will improve tumor region performance.

## 5. Conclusion

This study introduces T1C-RFlow, an efficient 3D latent diffusion model for synthesizing high-quality T1-contrast-like scans from multiparametric MRI for glioma, meningioma, and metastasis patients while making predictions in much less time than previous diffusion models. Our proposed method shows promising potential as a contrast-agent-free MRI alternative for imaging characterization of brain tumors, eliminating GBCA-associated risk and simplifying clinical protocols.

## Data Availability

The data that support the findings of this study are openly available at the following URL/DOI: https://www.synapse.org/Synapse:syn53708249/wiki/626323.
